# Combined Open Rhinoplasty and Vertical Incision Approach to Addressing a Recurrent Nasal Dermoid Sinus: A Case Report

**DOI:** 10.7759/cureus.77490

**Published:** 2025-01-15

**Authors:** David Wassef, Devanshi Patel, Arron Gravina, Brian Manzi

**Affiliations:** 1 Otolaryngology - Head and Neck Surgery, Rutgers University New Jersey Medical School, Newark, USA

**Keywords:** dermoid cysts, nasal endoscopy and excision, rhinology, rhinoplasty surgery, surgical approach

## Abstract

A three-year-old boy was referred to our clinic for a draining lesion on the dorsum of the nose, several months following the surgical excision of a nasal dermoid cyst in Egypt. Physical examination demonstrated a pit on the skin of the nasal dorsum, and magnetic resonance imaging (MRI) was consistent with dermoid cyst recurrence. The patient underwent reoperation using a combined vertical incision and open rhinoplasty approach, with excellent pathologic and cosmetic results. Here, we present this case as a surgical approach to consider in the excision of nasal dermoid cysts.

## Introduction

Dermoid cysts are benign cutaneous tumors that commonly present as a firm mass at birth or during early childhood. About 3% of all dermoid cysts occur in the nasal cavity, in the midline on the dorsum of the nose, between the columella and glabella [1,2]. They generally appear as a small, persistent or enlarging bump on the nose. A small hole in the skin that may leak milky fluid has also been observed. Dermoid cysts can commonly become infected, causing tenderness, warmth, and redness of the skin overlying the cyst. Nasal dermoid cysts are relatively rare, with a prevalence estimated to be 1 in 30,000 live births in the United States [3]. They generally arise during early embryonic development and are derived from ectodermal and mesodermal layers, and lined by stratified squamous epithelium [2]. They are also observed in association with various craniofacial deformities, including hemifacial microsomia, hypertelorism, craniosynostosis, cleft lip/palate, and auricular deformities. The pathogenesis is reasoned to be due to incomplete obliteration of neuroectoderm in the developing frontonasal area, leading to the formation of an isolated cyst or sinus tract [4]. Recurrences are uncommon and can be prevented by meticulous and complete excision of the primary lesion, which can be highly difficult in cases of recurrent infections of the cyst [5-8]. Surgical excision is regarded as the gold standard in managing nasal dermoid cysts, both in primary lesions and recurrences. Several surgical methods have been proposed, ranging from local excision to a combined intracranial-extracranial approach [6,9]. Commonly used techniques include a midline vertical incision, nasal endoscopy-assisted excision, and open rhinoplasty. A midline vertical incision has been previously described as being used in combination with nasal endoscopy [6,7,9-11]. However, to our knowledge, a combined approach of open rhinoplasty and vertical midline incision has not been commonly described in the literature. Here, we present this combined approach for the surgical excision of a recurrent nasal dermoid cyst in a three-year-old male patient.

## Case presentation

**Figure 1:**
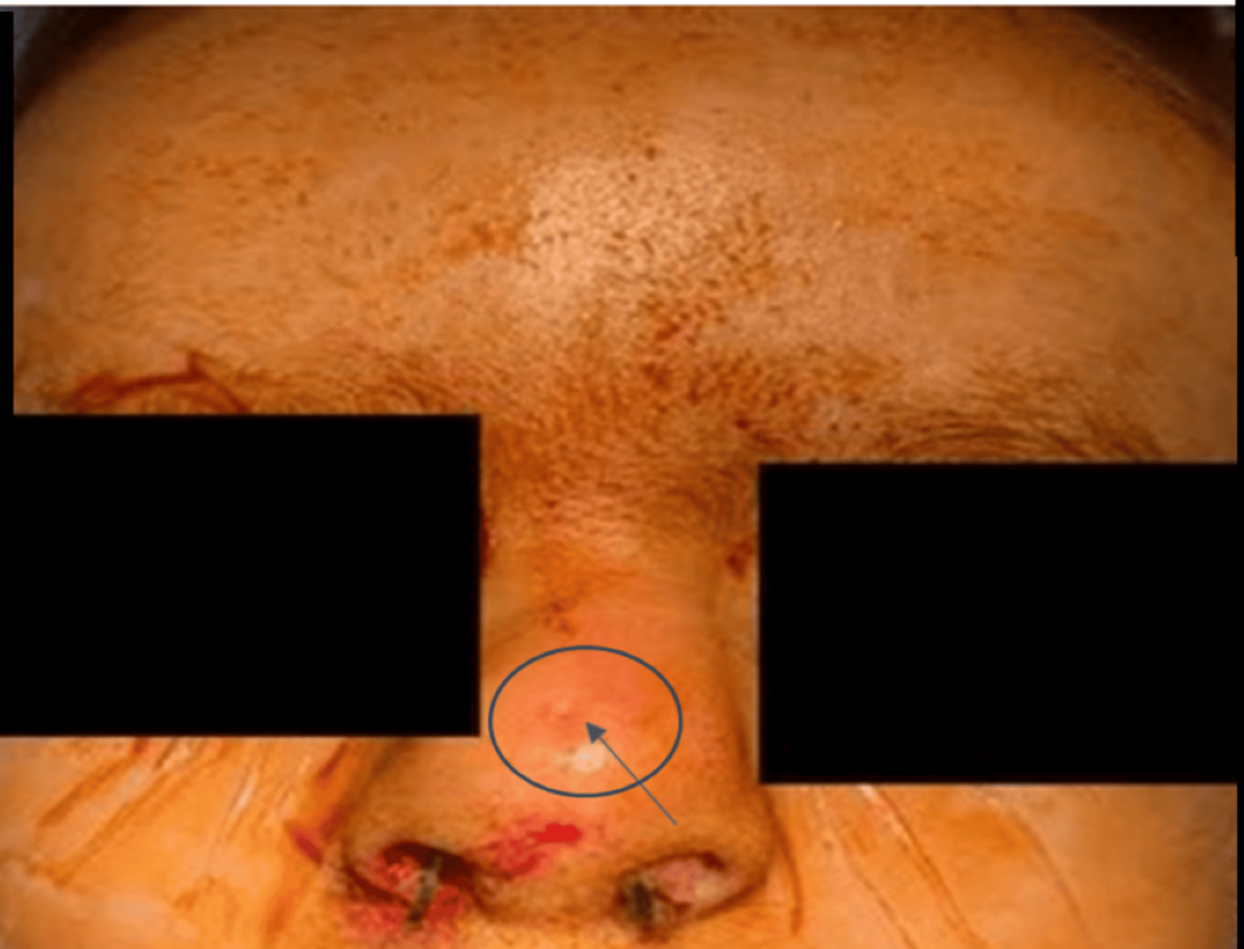
Intraoperative photo before incision demonstrating midline pit (arrow) indicative of nasal dermoid.

**Figure 2:**
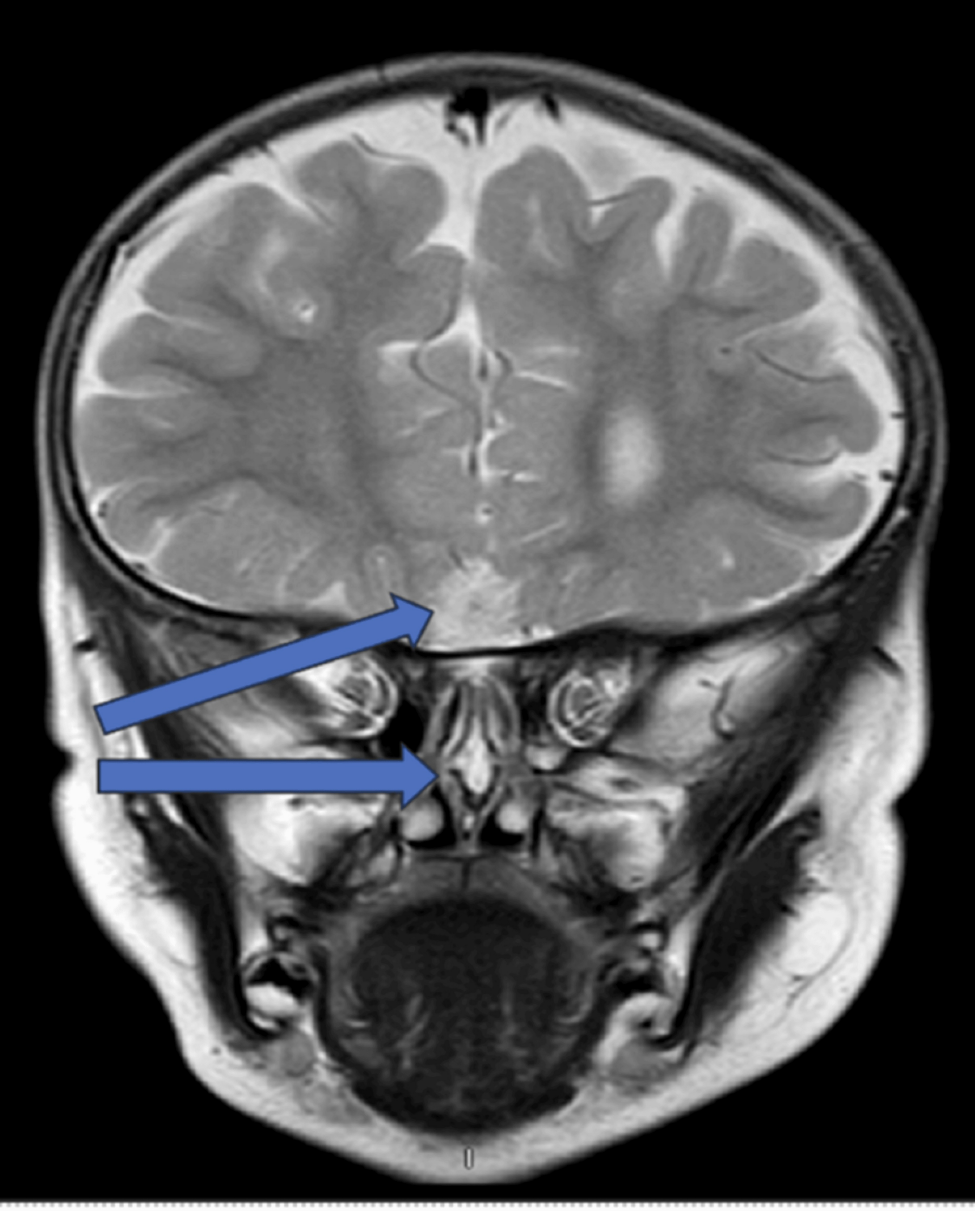
Coronal T2 weighted image demonstrating the cyst in the nasal cavity/septum as well as the separate intracranial lesion.

**Figure 3:**
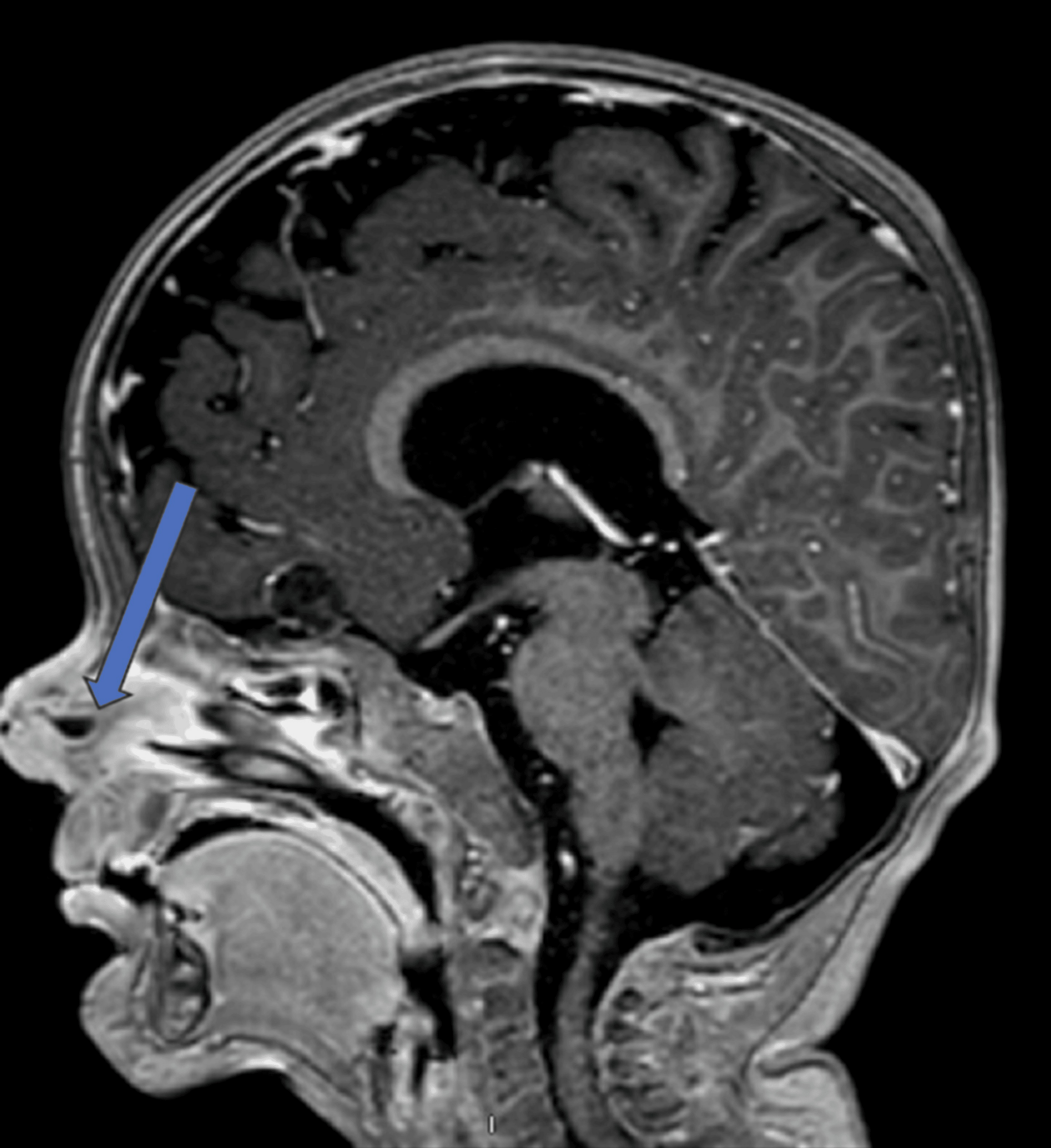
T1 sagittal post contrast MRI demonstrating blind ending of lesion. MRI, magnetic resonance imaging

A three-year-old male with a medical history significant for a horseshoe kidney and a nasal dorsal dermoid cyst that was removed via an open rhinoplasty approach at the age of 22 months, while the patient was living in Egypt. Prior records from Egypt were not available for review. On presentation to us in the clinic, the father reported that the patient was doing well for three to four months following surgery but then began to have intermittent purulent drainage from the nasal tip. Examination of the nose was notable for an external midline pit with surrounding erythema, but no active drainage (Figure 1) or any obvious obstructive intranasal lesions on the anterior rhinoscopy. A preoperative magnetic resonance imaging (MRI) was obtained, showing evidence of a residual midline nasal dermoid cyst without obvious intracranial extension. There was also a separate intracranial dermoid noted, which was not connected to the nasal component (Figures 2-3). Nasal endoscopy and operative excision of the recurrent nasal dermoid cyst were planned via a combined open rhinoplasty and vertical incision approach.

**Figure 4:**
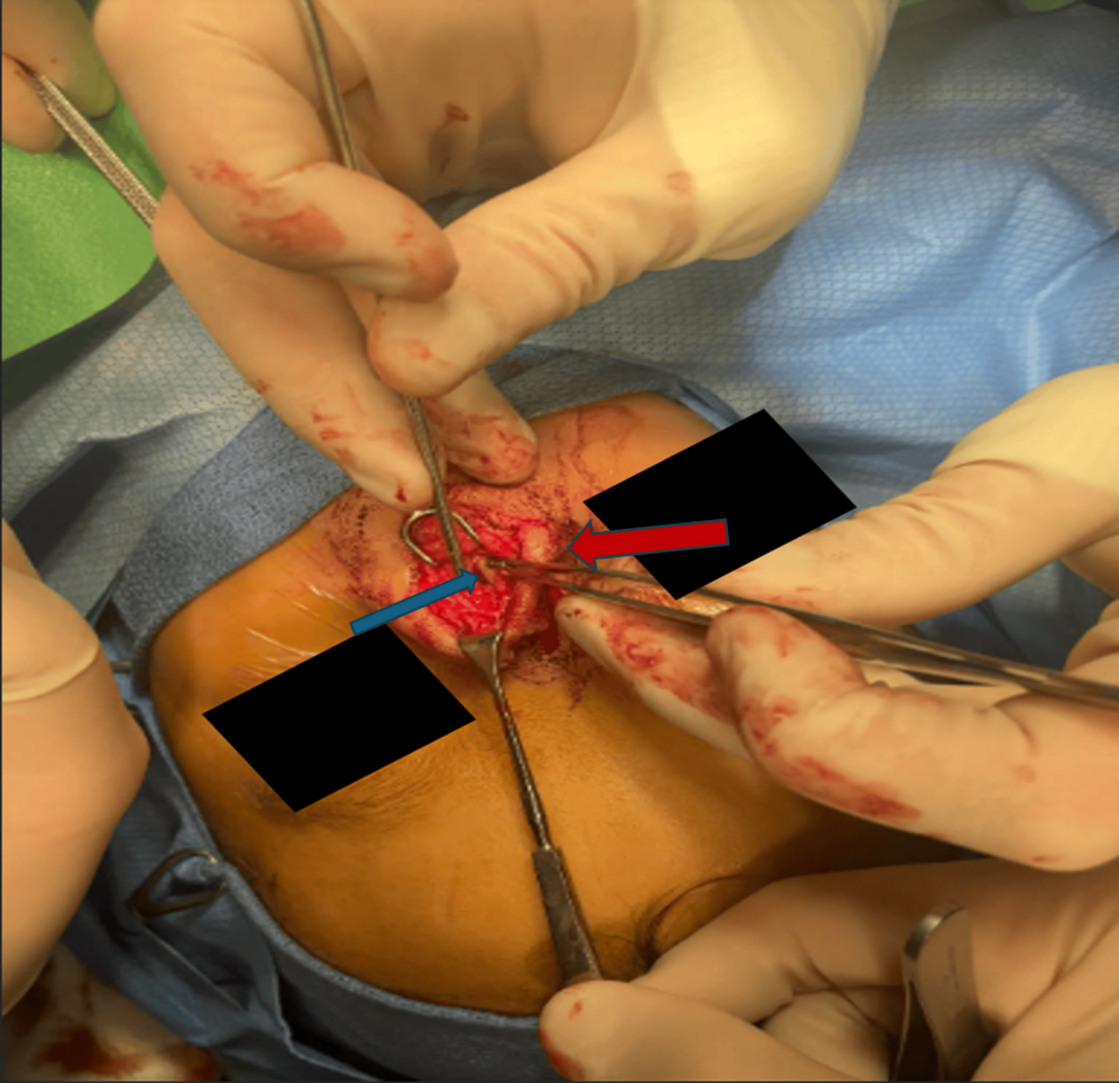
Isolating and dissecting the cystic tract (blue arrow) away from the nasal septum (red arrow).

**Figure 5:**
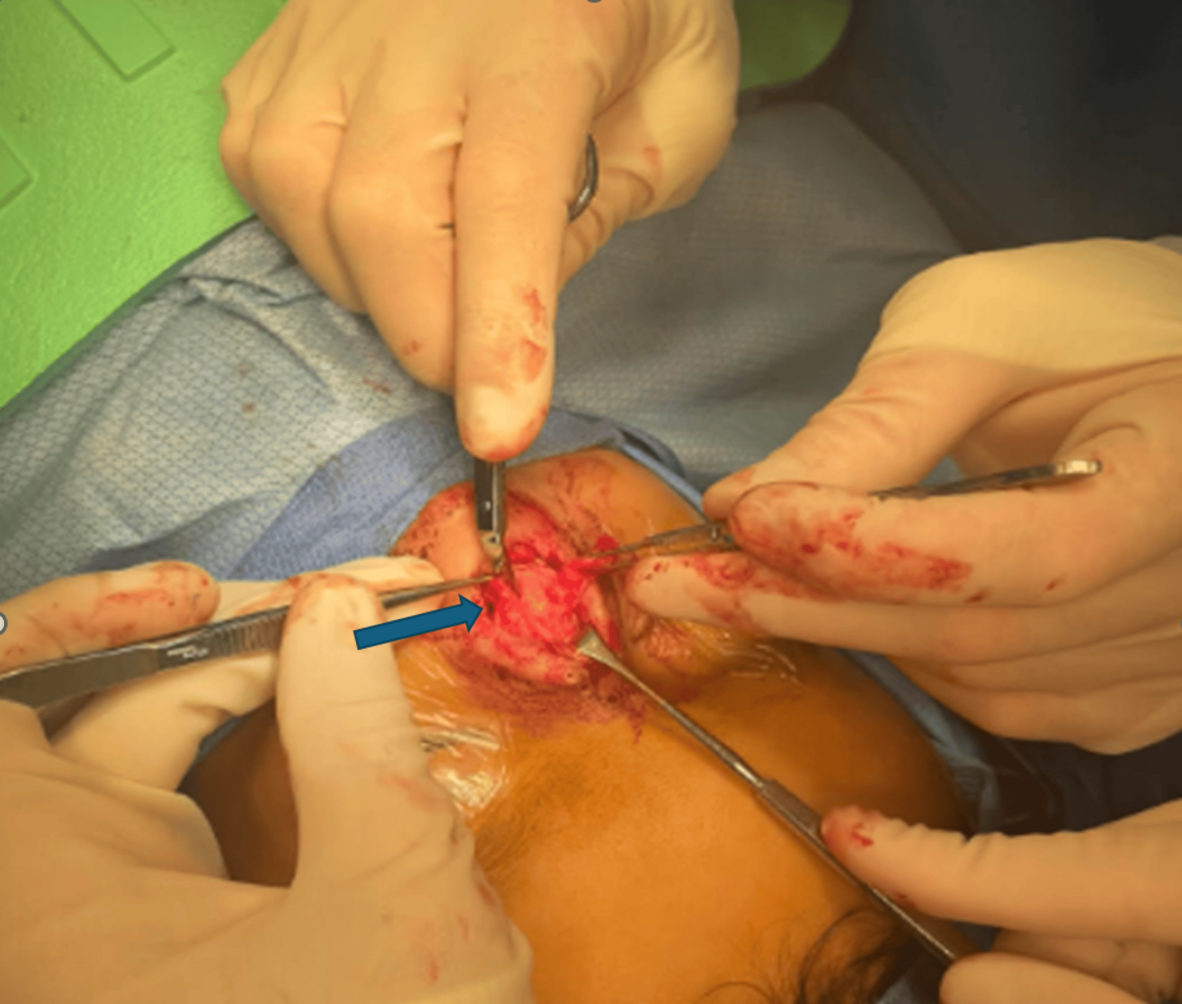
Dissection of the cyst and tract via the open rhinoplasty approach. The tract (blue arrow) is being retracted still attached to the dorsal dermal portion.

First, nasal endoscopy was performed bilaterally, which revealed normal-appearing mucosa without substantial distortion of normal anatomy and no obvious lesions. To start the exposure of the dermoid cyst, a vertically oriented elliptical incision was made over the dorsonasal lesion. We then made a V-shaped rhinoplasty incision in the line of his prior scar and elevated the skin envelope of the nose superior to the skin lesion, which was left in situ and separate from the rest of the skin envelope. The sinus cyst and tract were identified and delineated with blunt dissection, and the tract was noted to be diving into the septum, ending in a blind pouch. The lower lateral cartilages were divided with a #15 blade to expose the septal cartilage, which was further divided with a midline vertical incision to trace the sinus tract to a blind pouch in the middle of the septum (Figure 4). The cyst and tract were removed in their entirety, along with the diseased epidermis from the dorsum of the nose (Figure 5). The cartilage in the midline was closed, followed by the closure of the soft tissue. At the completion of the closure, a nasal endoscopy was repeated, noting the septum to remain intact and straight, with no perforations or mucosal defects. The final pathology of the mass identified a 1.8 x 1.0 x 0.7 cm mass that was consistent with a dermoid cyst.

Outcome and follow-up

**Figure 6:**
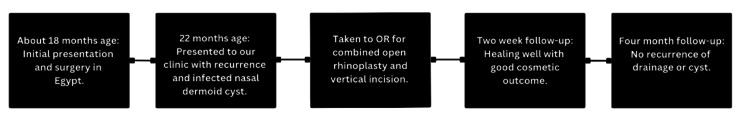
Timeline of events in patient presentation.

At a two-week follow-up, he was healing well, with excellent cosmetic outcomes and no loss of structural integrity of the nose. The patient also had a resolution of nasal drainage. He was seen again at a four-month follow-up, when he had no recurrence of drainage or sinus tract, and a fully healed, well-camouflaged nasal scar. Figure 6 shows a timeline of the patient’s initial presentation to four months post-op. 

## Discussion

**Figure 7:**
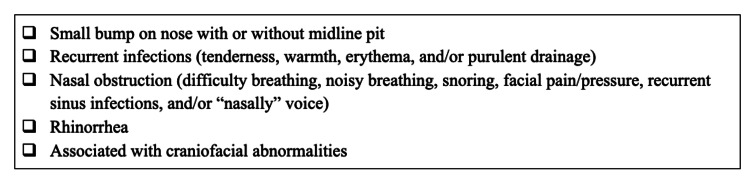
Symptoms of nasal dermoid cysts.

Nasal dermoid cysts are rare, benign cutaneous tumors commonly seen in the pediatric population, arising during early embryologic development due to incomplete obliteration of neuroectoderm in the developing frontonasal area [2-4]. Microscopic examination typically reveals a well-defined cyst lined by squamous epithelium of ectodermal origin, with adnexal structures of mesodermal origin [6]. They are also observed in association with various craniofacial deformities, although this was not seen in our case [4]. The most common symptoms of nasal dermoid cysts can be seen in Figure 7. Diagnosis usually occurs based on clinical presentation and imaging, generally with an MRI, which is also helpful in assessing the extent of the cyst and in ruling out intracranial extension [6,12]. Other differentials to consider can include encephalocele, ectopic neuroglioma, teratoma, and common epidermoid cysts. Appropriate management includes accurate diagnosis and treatment to avoid recurrence and complications, such as recurrent infections [2]. Although recurrences are rare, they can occur several years - about three to seven years - after initial surgery and are thought to be associated with incomplete primary excision. Therefore, long-term follow-up is essential for these patients [5-8,13]. This was the likely scenario for our patient, who had presented with a recurrence complicated by an infection at the time of presentation.

Surgical excision is regarded as the gold standard in management for primary dermoid cysts, as well as recurrences, with several surgical methods being proposed that range from local excision to a combined intracranial-extracranial approach, depending on the extension of the cyst and associated tract. Commonly used techniques include a midline vertical incision, nasal endoscopy-assisted excision, and open rhinoplasty [6,9,10].

The surgical approach should satisfy four criteria, including access to the midline cyst, access to the skull base, adequate exposure for the reconstruction of the nasal dorsum, and an acceptable scar post-resection [6]. Failure to adhere to these criteria is associated with unsatisfactory cosmetic and functional outcomes, with an increased risk of recurrence [10]. A midline vertical incision is the most commonly used approach, which allows satisfactory exposure of the entire cutaneous lesion and has adequate cosmetic results [6,10]. Nasal reconstruction to repair any defects can be achieved with local advancement flaps, regenerative tissue matrices such as AlloDerm, and bone dust plates with this technique. The midline vertical incision is the best option when encountering lesions with pits, or when there is a violation of the overlying skin [10]. Endoscopic techniques have been used in small lesions without significant cutaneous involvement and in combination with the vertical incision approach [7]. However, with these two techniques, there is a limitation in the exposure one can achieve, in comparison to open rhinoplasty. With open rhinoplasty, a wider exposure for the nasal osteotomy, easy access to follow the tract to the skull base, and a well-concealed scar, resulting in optimal cosmetic outcomes, can be achieved. Also, with this technique, there has been a lower rate of recurrences compared to the other techniques [6,11,13]. In a study of 42 patients, various approaches were compared, including vertical midline incision, external rhinoplasty, lynch incision, transverse incision, lateral rhinotomy, endoscopic technique, and combined approaches. In this case, external rhinoplasty was noted to have the best outcome, and the five recurrences they observed were spread out across all the different approaches [6].

In the current literature, there has been a preference for open rhinoplasty due to superior exposure and cosmesis. However, many of these studies did not comment on the extent of cutaneous involvement, which usually still benefits from a vertical excision approach [10]. This was our decision-making process behind the approach we used in our patient case. The vertical incision was made with the goal of excising the diseased skin in order to adequately address the cutaneous involvement. The addition of the open rhinoplasty approach offered broad exposure to fully visualize the cyst tract and surrounding deep nasal anatomy into the nasal septum. This allowed better visualization, which aided in delineating the lesion borders and allowed for separation from the surrounding nasal structures to help prevent recurrence, without sacrificing the structural integrity of the nose or cosmetic outcomes. We noted that this combined approach was especially helpful in this case of recurrence and repeated infections, where fibrosis can distort and obscure the usual tissue planes. This combined approach was observed to achieve optimal surgical and cosmetic outcomes at two-week and four-month follow-ups. Our experience also highlights the importance that surgical management of nasal dermoids should be determined on an individual basis, as in certain cases, a combined approach may prove to be more beneficial.

As our observation was limited to one pediatric case, there is space for future studies on a larger scale, with broader demographics and clinical trials, to determine the clinical significance of this combined approach. In addition, we were only able to follow up with our patient for up to four months; therefore, additional long-term studies would also be important. However, based on our experience, a combined approach of vertical incision and open rhinoplasty can be considered in cases complicated by recurrence and infection after a primary excision.

## Conclusions

Based on our experience, a combined approach using a vertical incision and open rhinoplasty can be considered for recurrent, infected nasal dermoid cysts, as it has the potential to produce favorable surgical and cosmetic outcomes.

## References

[1] Pinheiro-Neto CD, Snyderman CH, Fernandez-Miranda J, Gardner PA (2011). Endoscopic endonasal surgery for nasal dermoids. Otolaryngol Clin North Am.

[2] Hartley BE, Eze N, Trozzi M (2015). Nasal dermoids in children: a proposal for a new classification based on 103 cases at Great Ormond Street Hospital. Int J Pediatr Otorhinolaryngol.

[3] Paradis J, Koltai PJ (2015). Pediatric teratoma and dermoid cysts. Otolaryngol Clin North Am.

[4] Chu EA, Ishii LE (2010). Adult nasal dermoid sinus cyst. Ear Nose Throat J.

[5] Herrington H, Adil E, Moritz E, Robson C, Perez-Atayde A, Proctor M, Rahbar R (2016). Update on current evaluation and management of pediatric nasal dermoid. Laryngoscope.

[6] Rahbar R, Shah P, Mulliken JB (2003). The presentation and management of nasal dermoid: a 30-year experience. Arch Otolaryngol Head Neck Surg.

[7] Yang XJ, Zhang J, Tang LX, Wang PP, Sun JH, Wang YN, Ge WT (2020). Excision for congenital nasal dermoid and sinus cyst in children. Zhonghua Er Bi Yan Hou Tou Jing Wai Ke Za Zhi.

[8] Blake WE, Chow CW, Holmes AD, Meara JG (2006). Nasal dermoid sinus cysts: a retrospective review and discussion of investigation and management. Ann Plast Surg.

[9] Ni K, Li X, Zhao L, Wu J, Liu X, Shi H (2020). Diagnosis and treatment of congenital nasal dermoid and sinus cysts in 11 infants: a consort compliant study. Medicine (Baltimore).

[10] Ortlip T, Ambro BT, Pereira KD (2015). Midline approach to pediatric nasofrontal dermoid cysts. JAMA Otolaryngol Head Neck Surg.

[11] Makhdoom N, Abo El Ezz TA, Abdel-Haleem M (2017). Management of midline nasal dermoid lesions in children by external rhinoplasty. J Taibah Univ Med Sci.

[12] Naina P, Jonathan GE, Prabhakar M, Irodi A, Syed KA, John M, Varghese AM (2020). Pediatric nasal dermoid- a decade's experience from a South Indian tertiary care centre. Int J Pediatr Otorhinolaryngol.

[13] Bilkay U, Gundogan H, Ozek C, Tokat C, Gurler T, Songur E, Cagdas A (2001). Nasal dermoid sinus cysts and the role of open rhinoplasty. Ann Plast Surg.

